# Genome-Wide Analysis of the DUF1664 Family Genes in Peanut (*Arachis hypogaea*) and Functional Validation of *AhDUF1664-1A*

**DOI:** 10.3390/plants15071080

**Published:** 2026-04-01

**Authors:** Mingjing Zhang, Wenpeng Wang, Wei Wang, Xiaoping Wang, Qiuguo Shi, Shucai Wang, Siyu Chen, Shuxin Zhang, Xiaojun Hu

**Affiliations:** 1 Laboratory of Plant Molecular Genetics & Crop Gene Editing, School of Life Sciences, Linyi University, Linyi 276005, China; zhangmingjing@lyu.edu.cn (M.Z.);; 2 State Key Laboratory of Wheat Improvement, College of Life Sciences, Shandong Agricultural University, Tai’an 271018, China

**Keywords:** DUF1664, *Arachis hypogaea* L., salt tolerance, drought tolerance

## Abstract

The Domains of Unknown Functions 1664 (DUF1664) genes are a class of genes with unknown functions, and their roles in abiotic stresses responses have not yet been reported. Using the hidden Markov model (HMM) profile of DUF1664 (PF07889) obtained from the Pfam database, along with *Arabidopsis thaliana* DUF1664 family protein sequences as reference, and verifying complete DUF1664 domains with the NCBI CD-Search online tool, seven *DUF1664* family members were identified in the peanut (*Arachis hypogaea*) genome, designated as *AhDUF1664-1A* through *AhDUF1664-4*. Promoter analysis revealed that cis-acting elements in *AhDUF1664* genes are associated with growth and development, stress responses, and plant hormone signaling, and these genes exhibit relatively conserved motifs. Functional validation showed that ectopic expression of *AhDUF1664-1A* enhanced tolerance to salt and drought stresses in *Arabidopsis thaliana* by modulating the expression of ABA signaling-related genes. Our findings identify the *AhDUF1664* gene family in peanut and provide a basis for further investigation into the biological functions of these genes.

## 1. Introduction

Abiotic stresses, such as drought, high salinity, and extreme temperatures, are major environmental factors that limit crop productivity and ecosystem stability. These stresses progressively damage plants from the cellular to the whole-plant level through a series of cascading effects, including disruption of membrane structure, interference with photosynthesis and energy metabolism, and induction of oxidative damage [1]. At the cellular structural and functional level, abiotic stresses directly compromise the integrity of biological membrane systems [2]. Drought and salinity induce osmotic stress and cellular dehydration, altering the physical properties of membrane lipids and resulting in reduced membrane fluidity and increased permeability [3]. Meanwhile, abiotic stresses lead to excessive accumulation of reactive oxygen species (ROS) in plants, triggering oxidative stress [4,5]. This, in turn, causes membrane lipid peroxidation and impairs membrane protein function. As a result, intracellular electrolytes and organic solutes leak out, metabolic compartmentalization is disrupted, and cellular functions become severely impaired [6]. At the physiological and metabolic levels, water deficit and high salinity induce stomatal closure, restrict CO_2_ assimilation, and significantly inhibit photosynthesis [7,8]. Ultimately, these stresses inhibit root development, cause leaf wilting, sharply reduce biomass accumulation, and lead to pronounced declines in crop yield and quality [9].

Previous studies have demonstrated that mining stress-responsive genes from peanut and modulating their expression in plants can effectively enhance stress tolerance. For instance, heterologous expression of the peanut glutamyl-tRNA reductase gene *AhHEMA1* and the ferrochelatase gene *AhFC1* in tobacco specifically regulates the tetrapyrrole biosynthesis pathway, thereby alleviating oxidative stress-induced damage and protecting cell membranes under salt stress [10]. Heterologous expression of *AhLOX29* enhances reactive oxygen species (ROS) scavenging capacity in transgenic *Arabidopsis thaliana*, preserves membrane integrity, and consequently improves drought tolerance [11].

Similarly, heterologous expression of the peanut calmodulin gene *AhCaM4* enhances ROS scavenging capacity in transgenic tobacco, thereby increasing seed germination rates under salt stress. Its interacting protein, *AhSAMS1*, is also associated with plant tolerance to abiotic stress; transgenic *Arabidopsis* expressing *AhSAMS1* exhibits increased polyamine biosynthesis, enhanced ROS scavenging capacity, and improved salt tolerance [12,13,14]. Moreover, heterologous expression of the peanut MYB transcription factor gene *AhMYB30* elevates the expression levels of stress-responsive genes *RD29A*, *COR15A*, *KIN1*, and *ABI2* in *Arabidopsis*, thereby enhancing salt tolerance via the ABA signaling pathway [15]. Likewise, heterologous expression of *AhMYB44-11* in *Arabidopsis* upregulates the transcription of stress-related genes such as *AtP5CS1*, *AtRD29A*, and *AtCBF1*, promotes the accumulation of osmolytes including proline and soluble sugars, and improves drought tolerance [16]. In addition, heterologous expression of the peanut NAC transcription factor gene *AhNAC3* in tobacco activates the expression of *SOD*, *P5SC*, *LEA*, and *ERD10C*, thereby enhancing drought tolerance [17]. In the same way, heterologous expression of the peanut bHLH transcription factor gene *AhbHLH112* enhances ROS scavenging capacity in transgenic *Arabidopsis* and improves drought tolerance via an ABA-dependent pathway. Furthermore, *AhbHLH112* has been shown to directly bind to and activate the promoter of the *POD* gene, thereby regulating POD-mediated H_2_O_2_ homeostasis and contributing to tolerance against multiple abiotic stresses [18]. Collectively, these findings highlight that the identification and functional characterization of stress-responsive regulatory genes in peanut are crucial for enhancing tolerance to abiotic stresses and ensuring the productivity and food security of oil crops.

Domains of Unknown Function (DUFs) refer to a class of proteins with well-defined structural features but uncharacterized biological roles. In recent years, rapid advances in functional genomics and structural biology have led to increasing evidence that many DUF proteins play critical roles in plant life processes, including growth and development, stress responses, and metabolic regulation [19]. For instance, in rice, the DUF1001 family protein SSG7 interacts with key regulatory factors SSG4 and SSG6 to control starch granule size and is essential for endosperm development [20]. In soybean, *GmDUF4228* is required for flowering, reproductive success, and yield formation, while *GmDUF668* is involved in nitrogen metabolism and plays a key role in nitrogen fixation and legume development [21]. Overexpression of *OsDUF6* enhances the activities of antioxidant enzymes such as superoxide dismutase, peroxidase, and catalase, positively regulates Na^+^ transport, and thereby improves salt tolerance in rice [22]. Functional characterization of DUF proteins not only contributes to a more comprehensive understanding of plant biological processes but also provides new potential targets for crop genetic improvement.

Peanut (*Arachis hypogaea* L.) is a globally important oilseed and economic crop cultivated worldwide, providing a significant source of edible oil and high-quality plant protein for the global diet and food industry [23]. However, peanut growth and development throughout the entire life cycle are highly susceptible to multiple abiotic stresses, with soil salinity and drought being the two primary environmental constraints on yield and quality [24,25]. Salt and drought stresses suppress normal plant growth and development by disrupting physiological and metabolic processes [26]. Moreover, these stresses markedly affect the reproductive growth stage of peanut, leading to poor pod development, reduced pod number per plant, insufficient seed filling, and impaired oil synthesis and accumulation, ultimately compromising both economic yield and commercial quality [27,28]. Therefore, identifying novel regulators of abiotic stress responses, elucidating the physiological and molecular mechanisms underlying peanut responses to salt and drought, and applying this knowledge to develop stress-tolerant germplasm and optimize cultivation practices are of great significance for enhancing environmental resilience, ensuring stable and high yields, promoting sustainable development of the peanut industry, and safeguarding regional food security. Despite this importance, the role of *DUF1664* genes in peanut stress tolerance remains unexplored. In this study, based on the peanut genome database, we identified seven *DUF1664* genes in peanut and performed comprehensive bioinformatics analyses. By screening transcriptome data under hydroxyproline-simulated drought stress, we further identified a stress-responsive gene, *DUF1664-1A*, and conducted functional validation. Our results demonstrate that *AhDUF1664-1A* plays an important role in salt and drought tolerance. These findings provide a valuable theoretical foundation for further elucidating the molecular mechanisms of stress resistance in peanut and offer promising genetic resources for breeding stress-tolerant cultivars.

## 2. Results

### 2.1. Identification and Chromosomal Distribution of DUF1664 Genes in A. hypogaea

**Figure 1:**
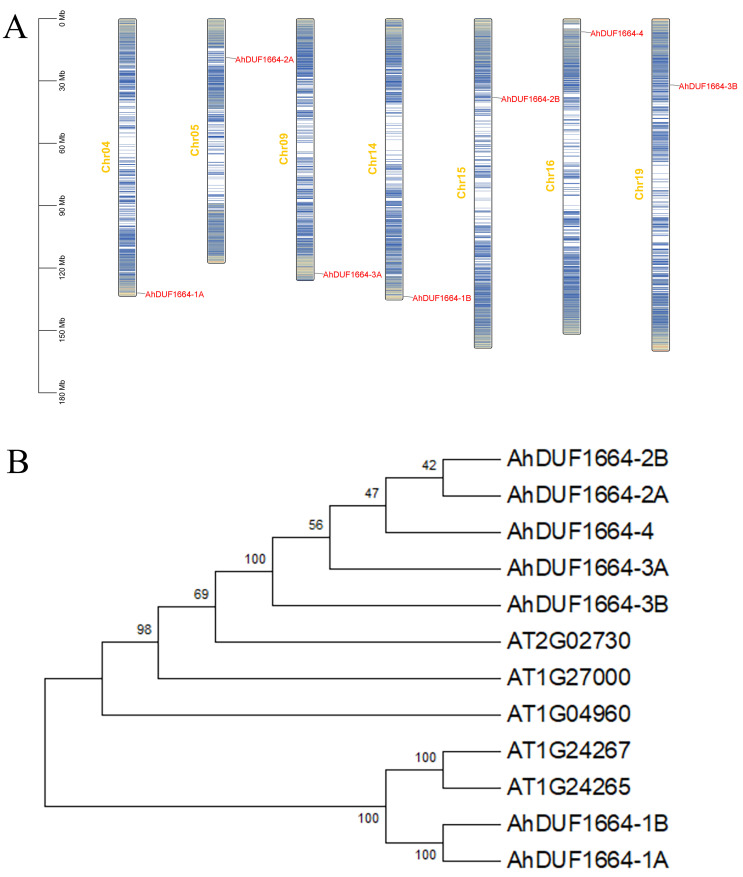
Chromosomal distribution (**A**) and phylogenetic tree classification (**B**) of *DUF1664* genes in *A. hypogaea*.

Using the HMMER model to search the peanut genome database and verifying complete DUF1664 domains with the NCBI CD-Search online tool, seven *DUF1664* genes were identified in the peanut genome. These genes were numbered according to their chromosomal locations and designated as *AhDUF1664-1A* to *AhDUF1664-4* (Figure 1A). The seven genes are distributed across different chromosomes. Among them, *AhDUF1664-1A* and *AhDUF1664-1B* possess the longest genomic lengths (1083 bp) and encode proteins of 360 amino acids, whereas *AhDUF1664-4* is the shortest (906 bp) and encodes a protein of 303 amino acids. The predicted molecular weights of these proteins range from 33,128.91 Da (AhDUF1664-4) to 38,578.76 Da (AhDUF1664-1A and AhDUF1664-1B). The isoelectric points of all AhDUF1664 proteins are below 10, ranging from 7.91 (AhDUF1664-3A) to 9.08 (AhDUF1664-3B) (Table S1). Evolutionary analysis indicates that, compared with *Arabidopsis*, the DUF1664 subfamily in peanut has likely undergone expansion. This expansion may be attributed to the gene redundancy arising from the allotetraploid origin of the peanut genome, together with species-specific gene duplication events, which collectively drove and maintained the enlargement of this subfamily.

### 2.2. Phylogenetic Tree Construction of AhDUF1664 Proteins

To clarify the phylogenetic relationships and evolutionary characteristics of the AhDUF1664 family proteins, a phylogenetic tree was constructed using the protein sequences of seven peanut DUF1664 members and five DUF1664 proteins from *Arabidopsis thaliana*. Based on the phylogenetic tree, the DUF1664 proteins from peanut and *Arabidopsis* are divided into two major groups. The first group comprises five peanut DUF1664 members and three *Arabidopsis* DUF1664 members, totaling eight proteins, whereas the second group contains four proteins (Figure 1B).

### 2.3. Structure and Motif Composition of AhDUF1664 Genes

**Figure 2:**
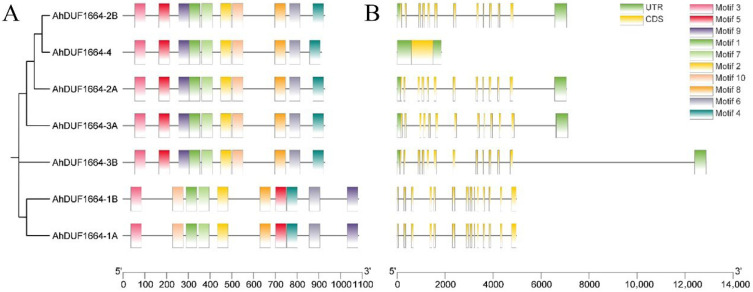
*DUF1664* genes’ structure and motif composition of *A. hypogaea*. (**A**) Motif composition of DUF1664 proteins in *A. hypogaea*; (**B**) Gene structure of *DUF1664* genes in *A. hypogaea*.

To investigate the sequence characteristics of the AhDUF1664 family, conserved motif analysis was performed using the MEME online tool. Ten conserved motifs, each 50 amino acids in length, were identified in all AhDUF1664 proteins (Table S3). Notably, except for *AhDUF1664-1A* and *AhDUF1664-1B*, the remaining five genes show identical motif positions (Figure 2A). This observation indicates that the *AhDUF1664* genes are highly conserved, and the motif distribution provides a structural basis for the phylogenetic analysis. Gene structure analysis shows that *AhDUF1664-4* contains only one exon, whereas all other *AhDUF1664* genes contain multiple exons (ranging from 12 to 14) (Figure 2B). The presence of multiple exons suggests that most peanut *AhDUF1664* genes may generate multiple splice variants with potentially diverse functions.

### 2.4. Promoter Analysis of AhDUF1664 Genes

**Figure 3:**
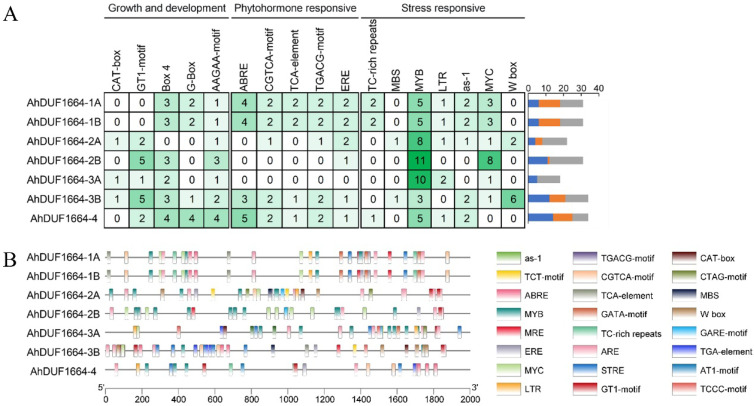
Cis-acting element analysis of *AhDUF1664* family genes. (**A**) The number of cis-acting elements in the promoter sequences of *AhDUF1664* family genes. The darker the color, the more cis-elements exist in the promoter sequence; (**B**) The distribution of cis-acting elements in the promoter sequences of *AhDUF1664* family genes.

Cis-acting elements form complex regulatory networks through specific interactions with corresponding trans-acting factors. Analysis of cis-acting elements in gene promoter regions enables the prediction of potential regulatory mechanisms and provides a molecular basis for understanding plant biological processes and improving stress tolerance. Therefore, the promoter sequences of *AhDUF1664* genes were analyzed using the PlantCARE online tool, revealing numerous cis-acting elements related to plant growth and development, stress responses, and hormone signaling. For example, G-box, CAT-box, and Box 4 are associated with the regulation of photosynthesis-related genes and photomorphogenesis and participate in plant growth and development; ABRE, TGACG-motif, and ERE are hormone-responsive elements involved in ABA, jasmonic acid, and ethylene signaling pathways; and MBS, W-box, and MYB-binding sites are related to plant stress responses. Analysis of typical cis-acting elements in *AhDUF1664* promoters shows that elements associated with growth and development are enriched in the promoter regions of *AhDUF1664-3B* and *AhDUF1664-4*. Numerous cis-acting elements related to abiotic stress responses are present in the promoters of *AhDUF1664-1A*, *AhDUF1664-1B*, and *AhDUF1664-2B*, whereas hormone-responsive elements are particularly abundant in the promoters of *AhDUF1664-1A*, *AhDUF1664-1B*, and *AhDUF1664-4* (Figure 3A). In addition to the aforementioned components, photoresponsive elements are distributed in the promoter regions of most members of the *AhDUF1664* family. In addition, some members’ promoters also contain cis-acting elements involved in tissue-specific expression or defense response (Figure 3B). These results indicate that the *AhDUF1664* gene family may participate in multiple biological processes, including plant growth and development, stress responses, and hormone signal transduction.

### 2.5. Expression and Subcellular Localization Analysis of AhDUF1664-1A

**Figure 4:**
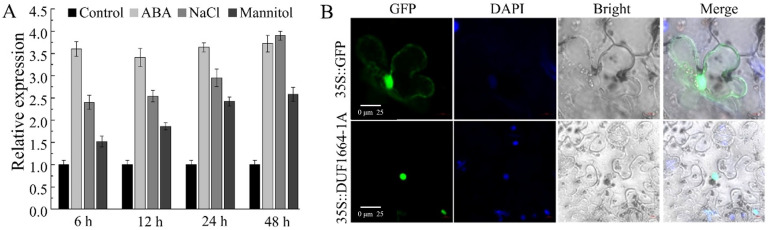
Analysis of *AhDUF1664-1A* gene characteristics. (**A**) Expression patterns under 200 mM NaCl, 300 mM mannitol and 10 μM ABA treatments; (**B**) subcellular localization of AhDUF1664-1A in leaf epidermal cells of tobacco, green fluorescence represents the GFP-tagged AhDUF1664-1A (or GFP alone), blue fluorescence denotes DAPI-stained nuclei, bright field shows cellular morphology, merge represents the overlay of fluorescent and bright-field images, scale bar = 20 µm.

To further investigate the response of peanut to environmental stresses, the expression levels of *AhDUF1664-1A* under ABA, mannitol, and NaCl treatments were analyzed by quantitative real-time PCR. The results show that *AhDUF1664-1A* responds rapidly to 10 μM ABA treatment, with a marked increase in expression at 6 h. Under 200 mM NaCl and 300 mM mannitol treatments, its expression gradually increases over time and peaks at 48 h (Figure 4A). These results indicate that *AhDUF1664-1A* acts as a positive regulator of ABA signaling and tolerance to drought and salt stress. To further characterize its function, an expression vector containing the *AhDUF1664-1A* coding sequence fused to GFP was constructed and introduced into tobacco leaves via agroinfiltration [29]. Subcellular localization was examined using laser scanning confocal microscopy. The GFP signal was predominantly detected in the nucleus, indicating that AhDUF1664-1A is localized to the nucleus (Figure 4B, Figure S1).

### 2.6. Eectopic Expression of AhDUF1664-1A Enhances Salt and Drought Tolerance in Arabidopsis thaliana

**Figure 5:**
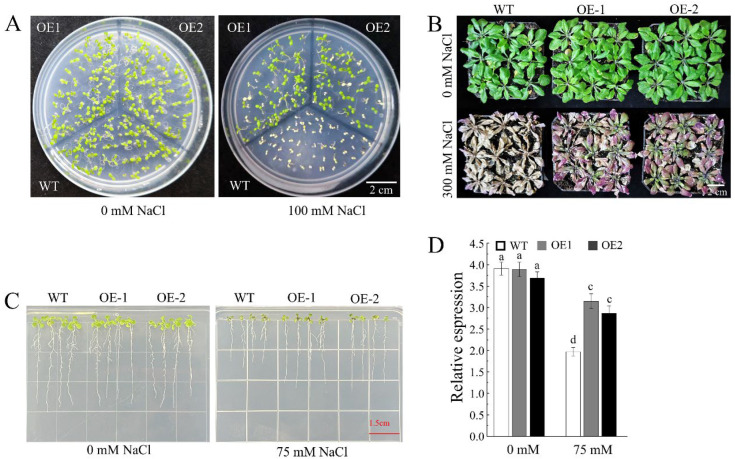
The phenotype of WT and ectopic expression of *AhDUF1664-1A* seedlings. (**A**) Seed germination phenotype of WT and ectopic expression of *AhDUF1664-1A* seedlings under 0 and 100 mM NaCl treatment. (**B**) Statistics of root length of WT and ectopic expression of *AhDUF1664-1A* under 0 and 75 mM NaCl treatment. (**C**) The phenotype of root length of WT and ectopic expression of *AhDUF1664-1A* seedlings under 0 and 75 mM NaCl treatment. (**D**) The phenotype of WT and overexpressing *AhDUF1664-1A* seedlings under 0 and 300 mM NaCl treatment, the lowercase letters a, c, d represent significantly different at *p* < 0.05 according to Duncan’s multiple range tests.

To investigate the function of *AhDUF1664-1A*, the *AhDUF1664-1A* gene was heterologously expressed in *Arabidopsis thaliana*. On 1/2 MS medium containing 100 mM NaCl, the survival rate of *AhDUF1664-1A* ectopic expression lines was higher than that of the wild-type (WT) (Figure 5A). Moreover, when grown on 1/2 MS medium supplemented with 75 mM NaCl, the root length of *AhDUF1664-1A* ectopic expression lines was significantly greater than that of WT plants (Figure 5B,C). Four-week-old seedlings of WT and transgenic lines, which exhibited consistent growth conditions, were subjected to salt treatment. After seven days of treatment with 300 mM NaCl, all plants showed growth inhibition and yellowing. However, the degree of wilting and yellowing in the transgenic lines was significantly lower than that in the WT plants (Figure 5D). These results indicate that the transgenic lines display improved tolerance and growth adaptability under high-salinity stress.

**Figure 6:**
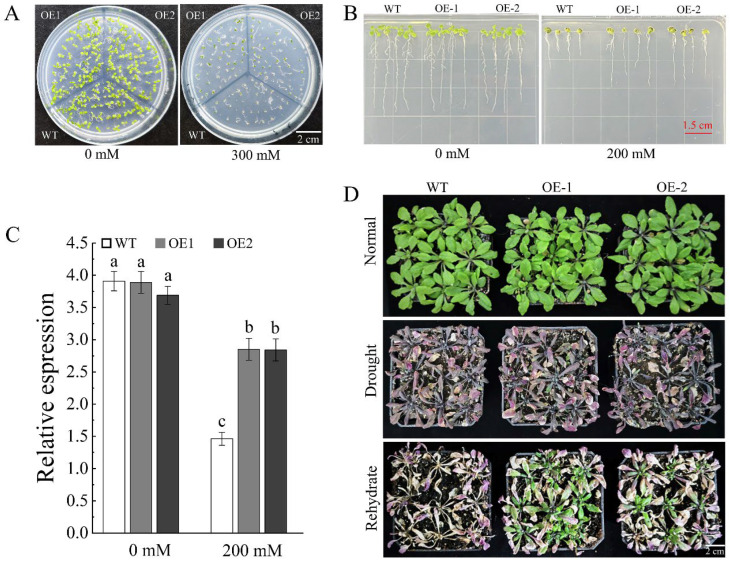
The phenotype of wild-type and ectopic expression of *AhDUF1664-1A* seedlings. (**A**) Seed germination phenotype of wild-type and ectopic expression of *AhDUF1664-1A* seedlings under 0 and 300 mM mannitol treatment. (**B**,**C**) The root length of wild-type and ectopic expression of *AhDUF1664-1A* seedlings under 0 and 200 mM mannitol treatment, and the lowercase letters a, b, c in subfigure (**C**) are significantly different at *p* < 0.05 according to Duncan’s multiple range tests. (**D**) The phenotype of wild-type and ectopic expression of *AhDUF1664-1A* seedlings under drought treatment.

To further explore whether *AhDUF1664-1A* also confers tolerance to osmotic/drought stress, we evaluated the performance of transgenic lines under mannitol-simulated drought conditions. Seeds of *AhDUF1664-1A* transgenic *Arabidopsis* were germinated on 1/2 MS medium containing 300 mM mannitol. Compared with WT, the transgenic lines show a significantly higher germination rate (Figure 6A). In addition, when grown on 1/2 MS medium supplemented with 200 mM mannitol, the root length of the ectopic expression lines was significantly greater than that of WT plants (Figure 6B,C). To further verify whether *AhDUF1664-1A* ectopic expression confers drought tolerance, 20-day-old seedlings were subjected to natural drought stress, followed by rewatering after 5 days of drought treatment. After 3 days of recovery, the transgenic plants exhibited a higher survival rate than WT plants (Figure 6B). These results demonstrate that, compared with WT plants, transgenic *Arabidopsis* shows improved adaptation to drought stress, and ectopic expression of *AhDUF1664-1A* enhances drought tolerance.

**Figure 7:**
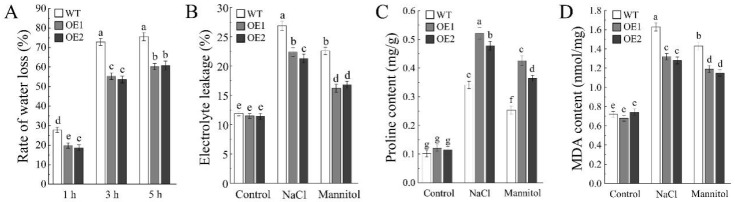
Physiological indicators of WT and ectopic expression of *AhDUF1664-1A Arabidopsis* seedlings under 200 mM NaCl and 300 mM mannitol treatment. (**A**) Water loss rate of wild-type and ectopic expression of *AhDUF1664-1A* seedlings under NaCl and mannitol treatment. (**B**) Electrolyte leakage of wild-type and ectopic expression of *AhDUF1664-1A* seedlings under NaCl and mannitol treatment. (**C**) Proline content of wild-type and ectopic expression of *AhDUF1664-1A* seedlings under NaCl and mannitol treatment. (**D**) MDA content of wild-type and ectopic expression of *AhDUF1664-1A* seedlings under NaCl and mannitol treatment. Values are the mean ± SD of three biological replicates. Bars with different letters are significantly different at *p* < 0.05 according to Duncan’s multiple range tests.

To further confirm the enhanced salt and drought tolerance of the transgenic plants, water loss rate, electrolyte leakage, proline content, and malondialdehyde (MDA) content were measured in WT and transgenic lines. Under drought conditions, the water loss rate of the ectopic expression lines was lower than that of WT plants (Figure 7A), indicating that *AhDUF1664-1A* ectopic expression enhances water retention capacity and reduces the rate of water loss. In addition, under both salt and drought treatments, electrolyte leakage in transgenic plants was significantly lower than that in WT plants (Figure 7B), suggesting that WT plants suffer more severe membrane damage, whereas transgenic plants maintain higher membrane stability and normal physiological status. Proline content showed no significant difference between transgenic and WT plants under normal conditions; however, after stress treatments, proline accumulation increased markedly in both genotypes, with significantly higher levels in transgenic plants than in WT plants (Figure 7C). This result indicates that transgenic plants accumulate more proline under salt and drought stress to mitigate stress-induced damage. MDA content increases significantly in both WT and transgenic plants after salt and drought treatments, but the levels in WT plants are markedly higher than those in transgenic plants (Figure 7D). These findings indicate that *AhDUF1664-1A* ectopic expression significantly enhances resistance to oxidative damage induced by salt and drought stress, thereby conferring greater salt and drought tolerance.

### 2.7. AhDUF1664-1A Enhances Salt and Drought Tolerance in Arabidopsis thaliana Through the ABA Signaling Pathway

**Figure 8:**
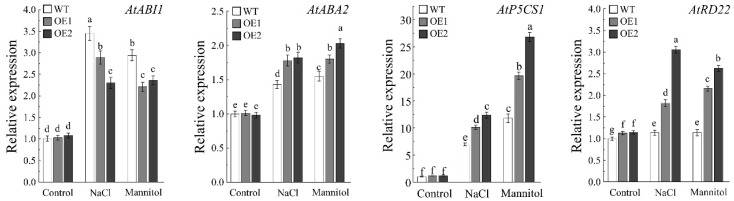
The expression of ABA signaling pathway-related genes in wild-type and transgenic *Arabidopsis* seedlings under 200 mM NaCl and 300 mM mannitol treatment. Values are the mean ± SD of three biological replicates. Bars with different letters are significantly different at *p* < 0.05 according to Duncan’s multiple range tests.

The abscisic acid (ABA) signaling pathway plays a central regulatory role in plant responses to abiotic stresses such as drought and salinity. As a key stress hormone, ABA enables plants to adapt to and survive adverse environments through a series of signaling and physiological responses. To elucidate the molecular mechanism by which *AhDUF1664-1A* regulates salt and drought stress responses, the expression of ABA pathway-related genes was analyzed by qRT-PCR in WT and *AhDUF1664-1A* transgenic *Arabidopsis* plants following salt and drought treatments. The results show that, compared with WT plants, the expression of the ABA biosynthetic enzyme gene *ABA2* is significantly increased in transgenic plants after 200 mM NaCl and 300 mM mannitol treatments, whereas the expression of *AtABI1*, a core negative regulator gene of the ABA signaling pathway, is markedly reduced. In addition, the expression levels of the stress-responsive genes *AtRD22* and *AtP5CS1* are significantly upregulated in transgenic plants (Figure 8). These results indicate that, under stress conditions, AhDUF1664-1A is closely associated with the ABA regulatory pathway and enhances the transcription of stress-responsive genes, thereby improving plant tolerance to salt and drought stress.

## 3. Discussion

DUFs (Domains of Unknown Function) represent a class of conserved protein domains or families whose biological functions have not yet been experimentally characterized [19]. These domains are considered important sources of innovation in plant adaptive evolution and the regulation of complex life processes. Elucidating the functions of DUFs contributes to understanding the molecular basis of plant cellular architecture, secondary metabolism, environmental perception, and signal transduction, and provides potential genetic resources for future molecular design-based breeding.

Systematic bioinformatic analysis of plant DUF gene families plays a pivotal role in functional genomics research. For example, twelve *DUF868* genes have been identified in tobacco, and analyses suggest that this gene family may participate in responses to drought, hormones, and other abiotic and biotic stresses. Overexpression of *NtDUF868-E5* promotes tobacco growth and development, enhances leaf photosynthetic capacity, and increases chlorophyll and carotenoid contents [30]. In rice, 69 *DUF247* genes have been identified, and expression analysis shows that, except for seven genes specifically expressed in panicles, most are constitutively expressed in seedlings, roots, stems, and leaves. Moreover, *DUF247* proteins contain transmembrane domains, suggesting important roles in rice development and environmental adaptation [31]. In ginger, twelve *ZoDUF668* genes have been identified, and bioinformatic analyses reveal that their promoters harbor multiple stress-responsive elements. Expression analyses demonstrate that these genes exhibit distinct expression patterns under *Fusarium* infection, low temperature, and water-logging stresses, providing candidate genes for improving stress resistance in ginger [32]. Compared with diploid plants, peanut, as a typical allotetraploid species, exhibits a pronounced paired distribution pattern of DUF1664 family members, reflecting gene duplication and divergence during genome doubling and rearrangement. This polyploid-specific gene redundancy provides a foundation for functional diversification, enabling certain genes to acquire novel regulatory roles or exhibit divergent expression patterns. In contrast, diploid plants such as *Arabidopsis* possess fewer DUF family members, with relatively limited functional diversification, typically displaying single-gene or small gene family-based regulatory modes.

Members of a gene family often share sequence similarity, yet their functions frequently diverge in a specific manner. Cis-acting elements in promoter regions function as “molecular switches” that determine gene expression patterns; thus, their analysis is central to explaining functional diversification among gene family members at the regulatory level. Identification of stress- and hormone-responsive elements in promoters provides strong mechanistic hypotheses for subsequent functional validation. Our analysis reveals that the promoters of peanut *AhDUF1664* genes contain numerous cis-acting elements associated with growth and development, hormone responses, and stress responses, including light-responsive elements such as the G-box and GT1 motif, ABA-responsive elements [33], and stress-responsive elements such as MBS and W-box. Compared with *AhDUF1664-2A* and *AhDUF1664-3A*, *AhDUF1664-1A*, *AhDUF1664-1B*, *AhDUF1664-2B*, *AhDUF1664-3B*, and *AhDUF1664-4* harbor a greater number of hormone-responsive cis-acting elements. Among the stress-responsive elements, MYB-binding sites account for the largest proportion, suggesting that these *AhDUF1664* genes may be regulated by MYB transcription factors. The core sequence of the G-box is typically CACGT and is widely present in promoters of light-responsive genes in plants. Notably, *Arabidopsis* ANAC060 specifically binds to the G-box element in the *ABI5* promoter and represses *ABI5* expression, thereby reducing sensitivity to sugar and ABA [34]. WRKY domains specifically recognize the core W-box sequence TTGAC [35]. OsWRKY5 directly binds to the W-box in the promoter of OsMYB2, repressing its expression and consequently downregulating *OsLEA3*, *OsRAB16A*, and *OsDREB2A*, which reduces drought tolerance in rice [36]. The MBS motif (CAACTG) is a specific binding site for MYB transcription factors; MdMYB2 directly binds to the MBS motif in the *MdSIZ1* promoter, activates *MdSIZ1* expression, and significantly enhances cold tolerance in apple [37]. MYC cis-acting elements are specific DNA sequences recognized by MYC transcription factors and primarily regulate genes involved in growth, development, and responses to environmental stresses [38,39]. By binding to these cis-elements, MYC transcription factors activate or repress downstream gene transcription, thereby influencing plant physiological processes.

In this study, *AhDUF1664-1A* is introduced into *Arabidopsis thaliana*. After NaCl and mannitol treatments, transgenic *Arabidopsis* exhibits significantly higher germination rates and longer root lengths than the wild-type. In addition, transgenic seedlings accumulate higher levels of proline and show lower malondialdehyde (MDA) contents compared with wild-type plants, indicating that ectopic expression of *AhDUF1664-1A* markedly enhances salt and drought tolerance. Proline accumulation contributes to maintaining osmotic balance within and outside cells and improves plant resistance to environmental stresses [40]. In soybean, *GmMYC2* reduces salt tolerance by regulating the expression of the proline biosynthesis-related gene *GmP5CS1* [39]. MDA content is directly associated with increased membrane permeability and electrolyte leakage and serves as an objective indicator of the degree of cellular membrane damage. Under stress conditions, increased proline accumulation facilitates timely scavenging of reactive oxygen species, leading to reduced MDA accumulation and enhanced stress tolerance. It has been reported that, compared with wild-type plants, transgenic *GmCOL1a* soybean exhibits higher relative leaf water content, increased proline levels, and lower MDA content, resulting in enhanced salt and drought tolerance [41]. Moreover, GmPM30-HapT interacts with GmLEA1 and GmLEC1 (lectin proteins), leading to significantly reduced ion leakage, MDA, and H_2_O_2_ contents under salt stress in HapT-type germplasm, thereby improving salt tolerance [42]. Further studies demonstrate that *BolMAM1* transgenic broccoli and *Arabidopsis* display stronger growth vigor, lower water loss rates and MDA contents, and significantly higher survival rates under salt and drought treatments [43]. In this study, heterologous expression of *AhDUF1664-1A* in *Arabidopsis* significantly enhanced plant tolerance to salt and osmotic stress. This finding is consistent with previous reports demonstrating that DUF proteins improve stress tolerance in other plant species, suggesting that this class of proteins may exhibit a certain degree of functional conservation across different species.

In summary, this study represents the first comprehensive investigation of the *AhDUF1664* gene family. We systematically analyze gene structures, conserved domains, evolutionary relationships, and promoter cis-acting elements of *AhDUF1664* genes. Furthermore, functional analyses of *AhDUF1664-1A* in transgenic *Arabidopsis* reveal its important role in enhancing drought and salt tolerance. These findings provide a foundation for further elucidation of the biological functions of *AhDUF1664* genes in peanut.

## 4. Materials and Methods

### 4.1. Plant Materials and Identification of DUF1664 Family Members

Peanut genome data were downloaded from the Figshare website (https://doi.org/10.6084/m9.figshare.26551309.v1). The hidden Markov model (HMM) profile of the DUF1664 domain (PF07889) was retrieved from the Pfam protein family database (http://pfam-legacy.xfam.org/, 28 February 2026). HMMER was used to search the peanut protein database for DUF1664 proteins, with the E-value threshold set to 1e−5 to obtain reliable DUF1664 protein sequences [44]. The NCBI CD-Search online tool was then employed to verify the presence of complete DUF1664 domains and to remove redundant sequences, thereby identifying the DUF1664 family members in peanut. Genes were named according to their chromosomal locations, and gene structure diagrams were generated using TBtools-II. The ProtParam tool (https://web.expasy.org/ protparam/, 28 February 2026) was used to calculate the physicochemical properties of the proteins. Conserved motifs were predicted using MEME (https://meme-suite.org/meme/index.html, 28 February 2026), and TBtools-II was used to visualize the gene structures and conserved motifs of the *DUF1664* gene family.

### 4.2. Multiple Sequence Alignment and Phylogenetic Analysis

Multiple sequence alignment of DUF1664 protein sequences from peanut and *Arabidopsis thaliana* was performed using MEGA7. A phylogenetic tree of the DUF1664 protein sequences from peanut and *Arabidopsis* was constructed using the neighbor-joining (NJ) method.

### 4.3. Promoter Cis-Element Analysis of DUF1664 Family Genes

The 2 kb genomic sequences upstream of the translation start codon (ATG) were defined as the promoter regions of *DUF1664* genes. The PlantCARE tool (https://bioinformatics.psb.ugent.be/webtools/plantcare/html/, 28 February 2026) was used to analyze these promoter sequences and to identify cis-acting elements potentially involved in growth, development, and stress responses.

### 4.4. Peanut Growth Conditions and Stress Treatments

Peanut seeds were germinated on MS medium in a growth chamber at 25 °C for two weeks. Subsequently, seedlings were subjected to stress treatments with 20% PEG 6000, 300 mM NaCl, 100 μM ABA, and low temperature. Leaf samples were collected at 0, 6, 12, 24, and 48 h after treatment for subsequent experiments. Each treatment included three biological replicates.

### 4.5. Generation and Treatment of Transgenic Arabidopsis thaliana Lines

The coding sequence of *DUF1664-1A* was inserted into the PZP expression vector using in-fusion cloning to generate a fusion expression construct. The construct was introduced into *Agrobacterium tumefaciens* strain GV3101 and subsequently transformed into *Arabidopsis thaliana* Col-0 plants using the floral dip method. Homozygous T3 transgenic *Arabidopsis* lines were used for further analyses.

After surface sterilization, wild-type and transgenic *Arabidopsis* seeds were evenly sown on 1/2 MS medium or on 1/2 MS medium supplemented with 75 mM NaCl, 100 mM NaCl, 200 mM mannitol, or 300 mM mannitol. Following stratification at 4 °C in the dark for 3 days, the plates were transferred to a growth chamber (23 °C/18 °C, day/night). Seed germination was monitored, and root length was measured after 7 days. In addition, 20-day-old *Arabidopsis* seedlings were irrigated with 300 mM NaCl, and phenotypic changes were recorded. For drought treatment, 20-day-old *Arabidopsis* plants were subjected to water withholding until complete leaf wilting, followed by rewatering; phenotypic changes were observed three days later.

### 4.6. Measurement of Physiological Parameters

Wild-type and transgenic *Arabidopsis thaliana* plants grown for 20 days were selected and divided into three groups. Each group was treated with Hoagland nutrient solution, Hoagland nutrient solution containing 200 mM NaCl, and drought treatment for 36 h before sampling for physiological index determination. Proline (A107-1-1) and malondialdehyde (MDA; A003-3-1) contents were determined using commercial assay kits (Nanjing Jiancheng Bioengineering Institute). Electrolyte leakage was measured according to the method described by [45], and the water loss rate was determined following the method of Zhang [46].

### 4.7. RT-qPCR Analysis

Gene-specific primers for ABA-responsive genes were designed using the Beacon Designer program (Table S2). RT-qPCR was performed on a Roche LightCycler^®^ 96 system (Roche, NY, USA). The RT-qPCR program consisted of initial denaturation at 95 °C for 3 min, followed by 40 cycles of 95 °C for 5 s, 55 °C for 15 s, and 72 °C for 20 s. Relative gene expression levels were calculated using the 2^−ΔΔCt^ method [47]. All experiments were performed with three biological replicates. The *ArabidopsisActin* gene was used as an internal control for data normalization and the calculation of relative expression levels.

## Data Availability

The original contributions presented in this study are included in the article/Supplementary Material. Further inquiries can be directed to the corresponding authors.

## Supplementary Materials

The following supporting information can be downloaded at https://www.mdpi.com/article/10.3390/plants15071080/s1, Figure S1: Heatmap of *AhDUF1664-1A* and other family members’ expression levels in different peanut tissues; Table S1: Analysis of physicochemical properties and subcellular localization prediction of AhDUF1664 proteins in *Arachis hypogaea*; Table S2: Primers used for fluorescence quantitative PCR of marker genes; Table S3: Base sequences of conserved motifs.
