# Supplementary material for: Genome-Wide Analysis of the DUF1664 Family Genes in Peanut (Arachis hypogaea) and Functional Validation of AhDUF1664-1A
Source: Plants (Basel). 2026 Apr 1;15(7):1080. doi: 10.3390/plants15071080 (PMC13074810; doi:10.3390/plants15071080)
Supplement: Supplementary file 1 [file plants-15-01080-s001.zip › Supplementary Table S1.pdf]

Supplementary Table S1 Analysis of physicochemical properties and subcellular localization prediction of AhDUF1664 proteins in *Arachis hypogaea*

Sequence ID	Number of Amino Acid	Molecular Weight	Theoretical pI	Instability Index	Aliphatic Index	Grand Average of Hydropathicity
AhDUF1664-1A	360	38578.76	8.93	50.34	87.50	-0.283
AhDUF1664-1B	360	38578.76	8.93	50.34	87.50	-0.283
AhDUF1664-2A	308	33687.55	8.59	35.69	95.68	-0.236
AhDUF1664-2B	308	33687.55	8.59	35.69	95.68	-0.236
AhDUF1664-3A	308	33668.50	7.91	36.46	95.68	-0.232
AhDUF1664-3B	308	33705.61	9.08	36.88	95.68	-0.241
AhDUF1664-4	303	33128.91	8.59	36.01	94.69	-0.243
