# Supplementary material for: Genome-Wide Analysis of the DUF1664 Family Genes in Peanut (Arachis hypogaea) and Functional Validation of AhDUF1664-1A
Source: Plants (Basel). 2026 Apr 1;15(7):1080. doi: 10.3390/plants15071080 (PMC13074810; doi:10.3390/plants15071080)
Supplement: Supplementary file 1 [file plants-15-01080-s001.zip › Supplementary Table S2.pdf]

Supplementary Table S2 Primers used for fluorescence quantitative PCR of marker genes

Primer name	sequence 5'-3'
AtABA1-F	GATTCTGGAGATAAGGTTACTGTGG
AtABA1-R	CCGTGTAACAAGTGTAGCCTGAAT
AtABA2-F	GCTTGTCTGTGTCAATAGTGAGGCA
AtABA2-R	GATCAGAGGAAAAAGTTGGAGAAAG
AtP5CS1-F	TCTTATGGCTTACTATGAGAC
AtP5CS1-R	TGGGGCTCTTCGGGTGCTAATAG
AtRD22-F	ATGATGACGACCGTCTACGC
AtRD22-R	ACATGAGTCTCCGGGAGGAA
