# Supplementary material for: Genome-Wide Analysis of the DUF1664 Family Genes in Peanut (Arachis hypogaea) and Functional Validation of AhDUF1664-1A
Source: Plants (Basel). 2026 Apr 1;15(7):1080. doi: 10.3390/plants15071080 (PMC13074810; doi:10.3390/plants15071080)
Supplement: Supplementary file 1 [file plants-15-01080-s001.zip › Supplementary Table S3.pdf]

Supplementary Table S3 Base sequences of conserved motifs

Motif name	Sequence
Motif 1	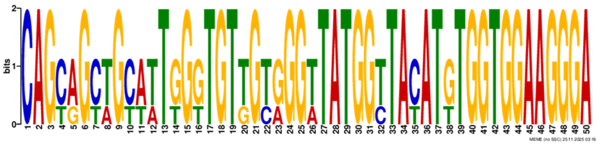
Motif 2	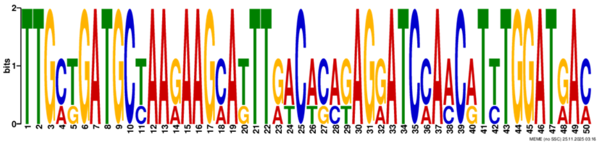
Motif 3	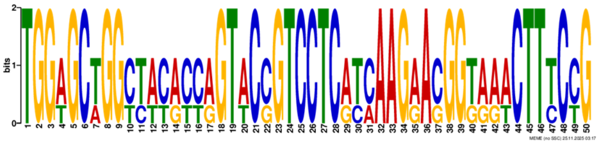
Motif 4	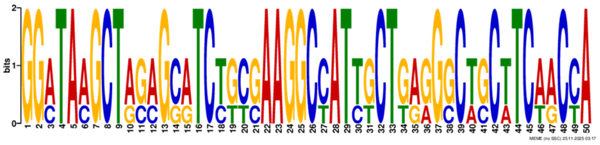
Motif 5	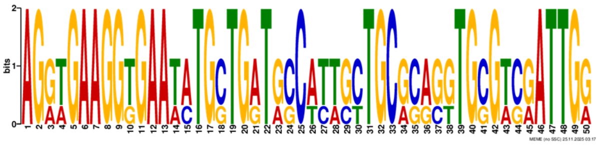
Motif 6	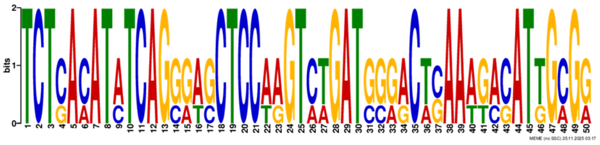
Motif 7	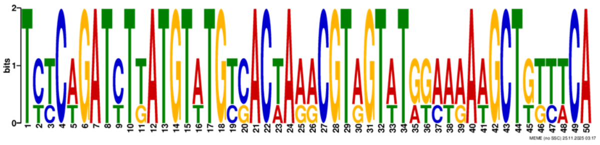
Motif 8	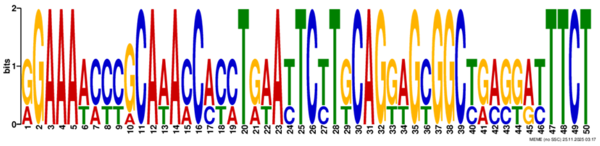
Motif 9	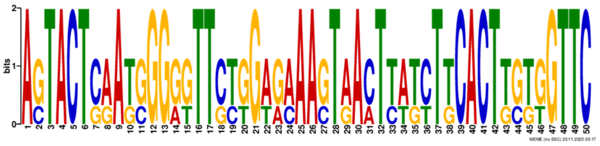
Motif 10	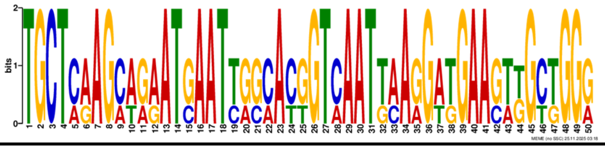
