# Supplementary figures and images for: Genome-Wide Analysis of the DUF1664 Family Genes in Peanut (Arachis hypogaea) and Functional Validation of AhDUF1664-1A
Source: Plants (Basel). 2026 Apr 1;15(7):1080. doi: 10.3390/plants15071080 (PMC13074810; doi:10.3390/plants15071080)

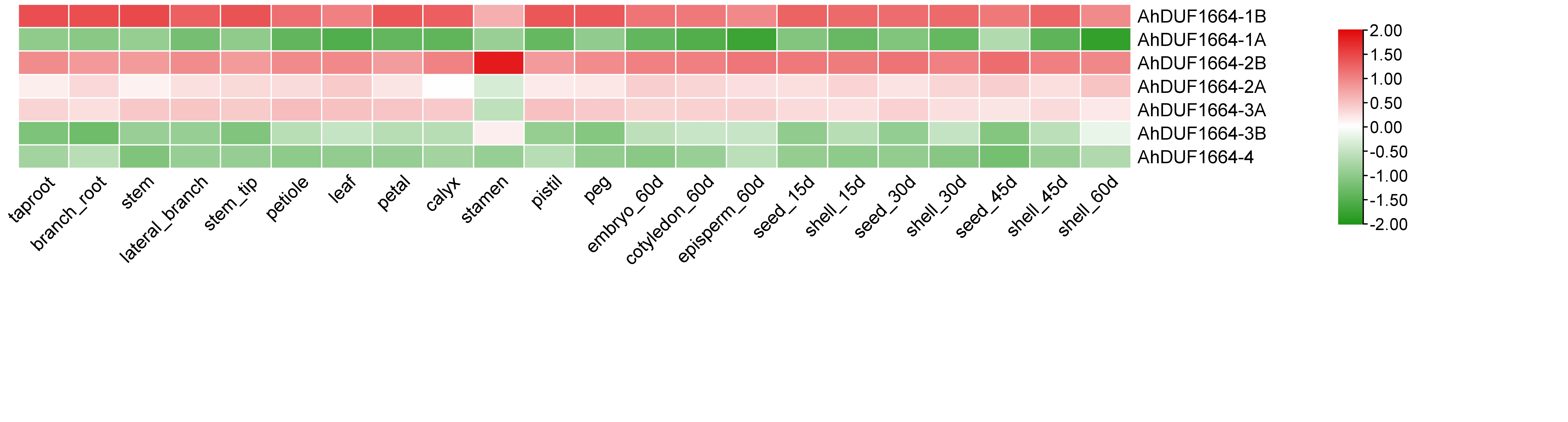

Supplement: Supplementary file 1 [file plants-15-01080-s001.zip › Supplementary Figure S1.jpg]
